# Temporal trends in equine sperm progressive motility: a systematic review and meta-regression

**DOI:** 10.1530/REP-22-0490

**Published:** 2023-05-02

**Authors:** I T Harris, C Maddock, M Farnworth, K Nankervis, J Perrett, A Z Pyatt, R N Blanchard

**Affiliations:** 1 HE Equine, Hartpury University and Hartpury College, Hartpury House, Gloucester, UK; 2 The Jeanne Marchig International Centre for Animal Welfare Education, The Royal (Dick) School of Veterinary Studies, Midlothian, UK; 3 International Office, Veterinary Medicines Directorate, Addlestone, UK; 4 School of Veterinary Medicine and Science, University of Nottingham, Sutton Bonington, Loughborough, UK

## Abstract

**In brief:**

Adverse trends in reproductive function are a concern in humans, companion, livestock, and wildlife species. This study indicates that equine populations are at risk of a comparable decline in sperm progressive motility.

**Abstract:**

There is increasing evidence reporting geographically sensitive adverse trends in human semen quality, with parallel trends observed in the dog sentinel. Despite significant economic and welfare complications associated with poor testicular function, trends in current equine populations are undetermined. Given the predictive value of sperm progressive motility (PMOT) in male factor infertility and fertilisation potential, research determining trends in this parameter is warranted. This research analysed trends in stallion sperm PMOT through systematic review and meta-regression. Using a comprehensive search strategy, Scopus, Embase (Ovid), Medline (Ovid), and VetMed (CAB direct) were scoped for eligible data. Using best practices, 230 meta-data points from 229 articles published from 1991 to 2021 were collated for meta-regression analysis. Sperm PMOT declined significantly between 1984 and 2019 (simple linear regression: *b* −0.340, *P* = 0.017; meta-regression: *b* −0.610, *P* ≤ 0.001). Overall and yearly PMOT declines were predicted at 33.51 and 0.96%, respectively (1984: 63.69 ± 5.07%; 2019: 42.35 ± 3.69%). Trends remained consistent irrespective of sensitivity analyses. Yearly and overall declines were stronger in western (yearly: 0.75%, overall: 26.29%) compared to non-western (yearly: 0.46%, overall: 10.65%) populations. Adverse trends contribute vital data to the debate surrounding declining semen quality, supporting the use of equines as novel comparative models for human reproduction. Results could have significant economic, health, and welfare consequences for equine breeding sectors. A comparable decline in human, dog, and horse sperm quality is indicative of a common environmental aetiology, indicating the need for a holistic One Health approach in determining causes and developing preventative strategies.

## Introduction

Research in human populations presents an increasing body of published evidence indicating that human reproductive health and function have declined over the last 40–60 years. Adverse trends in semen quality became a topic of contentious debate in 1992, initiated by a systematic review and meta-analysis reporting a significant decline in human sperm count from 1940 to 1990 (Carlsen *et al.* 1992). Results were heavily scrutinised due to heterogeneity, the inclusion of data from historical sources, and advancing semen analysis methods (Fisch 2008, Pacey 2013). The study operated as a stimulus for research, furthering the debate surrounding trends (Fisch 2008), with reanalyses supporting declines in semen quality (Swan *et al.* 2000, Johnson *et al.* 2015, Levine *et al.* 2017, Levine *et al.* 2022). Between 1973 and 2011, declines of 52.4 and 59.3% were reported in sperm concentration and total sperm count, respectively, with trends being more significant in Europe, North America, Australia, and New Zealand, compared to South Africa, Asia, and South America (Levine *et al.* 2017). Updates to the analysis continue to indicate a decline (Levine *et al.* 2022), raising concern over the reproductive health of human populations.

Despite evidence of declining semen quality in men, results remain a heavily debated topic (Tong *et al.* 2022), with comparative species required to contribute vital understanding towards trends (Wahl & Reif 2009, Lea *et al.* 2016, Sumner *et al.* 2020). Currently, research focuses on the dog sentinel, with a limited exploration into the suitability of the equine species as a comparative model. Retrospective research indicates a 30% decline in sperm motility between 1988 and 2014 in the dog sentinel model (Lea *et al.* 2016), with adverse trends continuing from 2014 to 2020 (Harris *et al.* 2022). In Holstein dairy bulls in the USA, seminal volume, sperm concentration, total daily sperm output, and normal morphology declined between 1965 and 1995 (Wahl & Reif 2009). Other research on bulls (Setchell 1997, van Os *et al.* 1997), pigs, and sheep indicate no decline towards the end of the 20th century (Setchell 1997). In equines specifically, research determining trends in sperm quality is inconclusive and unrepresentative of current trends or wider populations, limiting the applicability of the results (Multigner *et al.* 1999, 2000).

Current evidence on syntheses do not explore temporal trends in motion characteristics in any species. Sperm progressive motility (PMOT) is an important parameter for the prediction of male infertility (Dcunha *et al.* 2022), essential for the penetration of the zona pellucida and fertilisation potential (Simon & Lewis 2011). PMOT is a predictive parameter that can be utilised to monitor trends in reproductive health and function. Within the equine industry, threshold PMOT values have been proposed for fertility prediction (Parlevliet *et al.* 1994, Colenbrander *et al.* 2003, Aurich *et al.* 2020). Readings below proposed thresholds have been reported in stallions throughout the literature including those defined as fertile (Florez-Rodriguez *et al.* 2014, Cabrera *et al.* 2018, Ortiz-Rodriguez *et al.* 2019, Griffin *et al.* 2020). Suboptimal stallion semen quality is of significant concern for the breeding sector. Further research elucidating temporal trends is vital to initiate a holistic approach to the protection of equine reproductive health and function.

In testing the hypothesis of declining equine semen quality, these data could initiate the utilisation of this species as an additional comparative model for human fertility. Determining trends in stallion semen quality could therefore support or contradict previous research in other species, adding significant weight to the debate surrounding declining reproductive parameters. This evidence aimed to evaluate trends in sperm PMOT across global domesticated equine populations, to determine the current reproductive status and initiate the equine model as a novel comparative species for human reproduction.

## Methods

Hartpury University Ethics Committee granted ethical acceptance (ETHICS2019-52). This systematic review and meta-regression was conducted according to recommendations outlined by the Collaboration for Environmental Evidence (CEE 2018) and RepOrting standards for Systematic Evidence Syntheses (ROSES) (Haddaway *et al.* 2018). A qualified librarian, subject, and methodological specialists formed the research group. NVivo (QSR International version 12) was utilised as management and screening software for all stages of this review. A predefined protocol, including a test-search, literature scope, and a test-list, informed the development of this review (CEE 2018). The test-list, defined as a group of articles deemed relevant to answer the specific research question (CEE 2018), acted as a pilot study, and involved screening the title and abstract, and full texts of 50 and 38 articles, respectively.

### Systematic review

Between January and May 2021, Scopus, Medline (Ovid), Embase (Ovid), CAB direct (VetMed), and CORE were searched for English-language-only articles. Databases were selected based on results from literature scoping in the pilot study and previous recommendations (Grindlay *et al.* 2012, Bramer *et al.* 2017, Gusenbauer & Haddaway 2020). A Boolean search string was used to search the title and abstract of returned articles: (stallion* OR horse* OR equine* OR colt*) AND (sperm* OR (semen AND quality) OR insemin*) AND NOT (human* OR horseradish). Original articles retrieved from the implementation of the same search strategy for an alternative subset of databases (BASE, PubMed, PubAg) were included within the study (Perrett *et al.* 2021), following the same extensive review process reported within this review. The review considered all articles reporting data on fresh equine sperm PMOT) assessed objectively via computer-assisted semen analysis (CASA). The review also included fresh and cooled PMOT values analysed through microscopy, and sperm concentration analysed via a NucleoCounter, although these parameters were removed from the dataset following narrative synthesis due to the volume of returns and subjectivity in analysis methods.

Article screening involved three independent reviewers (ITH, CM, and JP) based on the predefined eligibility criterion (Table 1). Inconsistencies in opinions on article eligibility were resolved through group discussions with the research team. Article eligibility was determined through a two-tiered process: screening the title and abstract and full text (stage two). Supplementary information collected from citations, and through contact with corresponding authors for missing article data, were used to determine eligibility where relevant. Whilst the test group was not always suitable for inclusion, the control population was considered. Data from grey literature were excluded if duplicated in published formats. Review articles were not included within the final meta-regression, although were accepted through stage one to be included in citation searching. Articles eligible for inclusion at stage two were subject to forward (FC) and backward citation (BC) screening. The FC search was carried out using the Google Scholar platform, whilst BC searching involved screening the reference list of each article.

**Table 1:** Eligibility criteria separated into study-specific, population, and outcome components.

Question elements	Eligibility criteria	
	Inclusion	Exclusion
Study-specific	English language documents (original and translated)	Publications in a language other than English or without translation
	Peer-reviewed published literature including primary and retrospective data and case reports	Data presented in review or opinion articles
	Academic grey literature including dissertations and theses, conference presentations, and posters	Duplicate data sets
Population	Domesticated *Equus caballus* only	Alternative sub-species of the *Equus* genus
Outcome	Standard methods of semen collection including an artificial vagina	Non-standard semen collection methods including epididymal retrieval
	Semen quality analysis of PMOT and sperm concentration	Semen quality parameters presented for sexed semen samples
	PMOT values analysed via CASA or microscopy	Ejaculatory stimulation via pharmacological- and electro-methods
	Sperm concentration analysis via the NucleoCounter	Stallions displaying signs of perturbed reproductive health including anatomical, seminal, and bacterial or poor libido
	Fresh and cooled semen samples	Parameters reported for cryopreserved or frozen-thawed semen

### Critical appraisal and bias assessment

The implemented methodological and statistical approaches sought to mitigate bias at all stages. As a final method of bias assessment, all included studies were critically appraised in line with the CEE recommendations (CEE 2018). Each article was assessed based on five domains of bias (selection, performance, detection, attrition, reporting and other) and defined as low or high risk. High-risk articles for any domain were excluded from the dataset. Two independent reviewers (ITH and CM) critically appraised all relevant articles following the same method of assessment. Any articles with eligibility discrepancies were removed from the dataset.

### Data extraction

Summary statistics (mean, s.d., s.e.m., range, median, interquartile range) on PMOT and concentration were extracted from eligible articles. Data were extracted on sample collection year and sample size with covariates including fertility, location, season, breed, semen form (fresh or cooled), temperature and time cooled, extenders and centrifugation, method of analysis, CASA model, straightness (STR) and velocity average path (VAP) thresholds.

For missing key variables, estimations were made where possible. For studies that reported the mean and s.d. or s.e.m. in graphical formats, ImageJ (Image Processing and Analysis, Java) software was used to calculate estimations for each value. Where studies provided raw data or mean values on an individual stallion basis, an overall mean was calculated, resulting in a single value per article. Each article was given a label to define the method of data collection that occurred: graph extraction, calculation, or given value. For studies that failed to provide a sample collection year, the value was estimated by subtracting the mean difference between the publication year and collection year in studies that provided both values (Levine *et al.* 2017). In the instance where a study reported the s.d. but not the s.e.m. of PMOT, the s.d. was divided by the square root of the sample size for each estimate to calculate the s.e.m. When neither s.d. nor s.e.m. was reported, the mean s.d. of studies that reported this value was divided by the square root of the sample size to estimate the s.e.m. (Levine *et al.* 2017). When threshold values for CASA settings were given for total motility and not PMOT, the mean difference between values in studies that reported both were added to the motility values to calculate threshold CASA setting values for PMOT. Where a sample size was not provided, it was estimated that each stallion provided a single sample. For studies that failed to state the country of sample location, the author affiliations associated with the publication were used.

### Narrative synthesis

All data associated with concentration (*n* = 3), microscopy PMOT values (*n* = 22), and cooled semen (*n* = 30) were removed from the dataset. This resulted in 229 original datasets on fresh PMOT values analysed objectively via CASA. Stallions were defined as fertile or unselected for fertility. Covariates, including breed, season, hemisphere, and CASA model were grouped accordingly using established methods. The season was categorised as breeding, non-breeding, or year-round, in reference to hemisphere. Equine populations were defined as based in western (Europe, North America, and Australasia) and non-western (South America, Africa, and Asia) regions.

### Statistical analysis

Mean PMOT estimates for each eligible study were evaluated to model trends across time, as assessed by the predicted overall and yearly percentage changes and the slope of regression (*b)*. A simple linear regression model, weighted by study sample size, was run to assess time trends in PMOT. Mean (± s.e.m.) values were predicted from the model for each year of collection to determine overall trends. Following statistical guidance, a fixed-effects meta-regression with a stepwise approach was utilised to determine the significance of covariates, which were accounted for in the model if *P* < 0.05. Regardless of significance, the collection year was included. An inverse weighting metric was utilised for analysis. Utilising inverse variance allows larger studies to be given more weight than smaller studies, minimising potential uncertainty within the data, which is often a limitation of meta-analytical studies (Thompson & Higgins 2002). Accounting for covariates, the collection year was dropped from the model to determine its significance. Cubic and quadratic functions were added to the collection year in the model to assess non-linearity.

Multiple sensitivity analyses were performed to evaluate trends in relation to different contexts and populations. Sensitivity analyses included a meta-regression model weighted by study s.e.m., initially containing all predefined covariates. The significance of covariates was evaluated for each individual sensitivity analysis to determine the final fixed model. Sequential meta-regression analyses were run, excluding studies with less than a sample size of 10 (Borenstein *et al.* 2009), datasets with missing values for any variable, and articles before the collection year 2000. Groups of stallions ‘unselected for fertility’ (*n* = 152) were analysed to evaluate trends relevant to wider populations. Groups belonging to western (*n* = 191) and non-western (*n* = 32) regions were analysed separately to determine geographical differences. A single value was calculated for all articles, apart from one, which provided two values. To assess the impact of this article on the dataset, the meta-regression was run sequentially, removing each value followed by the article altogether. A *P* value of <0.05 was considered statistically significant, whilst a *P* value of <0.001 was highly significant and a 95% confidence interval (CI) was assumed for all analyses.

## Results

### Systematic review output

**Figure 1:**
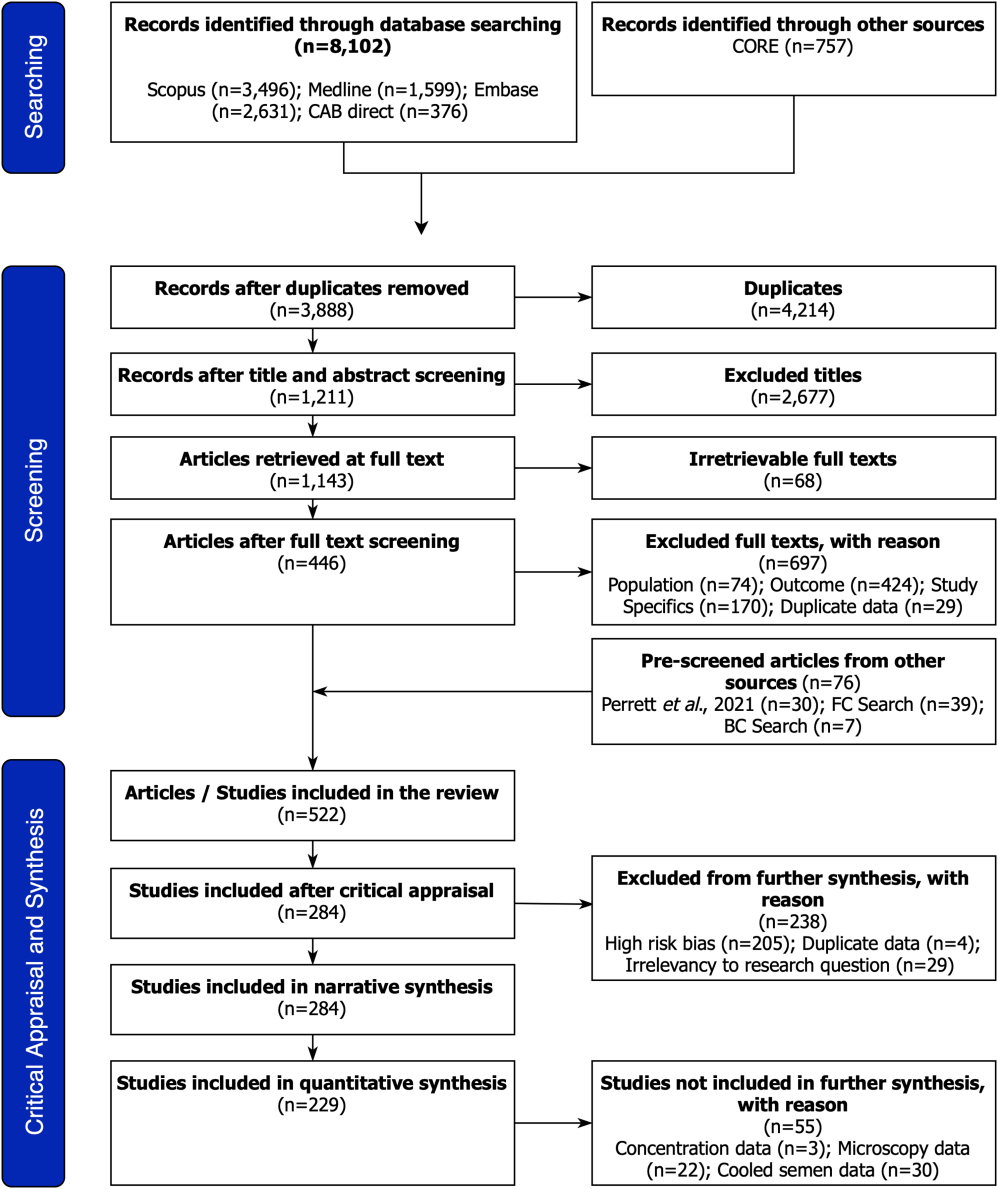
ROSES flow diagram for systematic reviews (Version 1.0) indicative of the searching, screening, and critical appraisal of articles (Haddaway *et al.* 2017). BC, backward citation search; FC, forward citation search; *n*, number of articles.

Using Scopus, Medline (Ovid), Embase (Ovid), and VetMed (CAB direct), 8102 publications were identified. A further 757 grey literature articles were identified (CORE). Of these, 4214 duplicates were excluded. Following article screening, 2677 articles were removed during the title and abstract stage and 697 were excluded after full-text screening. The full texts for 68 articles were not available and unobtainable through library resources. An additional 76 articles were accepted through stage two screening from FC and BC searching and alternative databases (Perrett *et al.* 2021). During the critical appraisal, 238 articles were excluded, resulting in 284 original articles (Fig. 1) (Haddaway *et al.* 2017).

### Narrative synthesis

The final dataset consisted of 229 original articles from publication dates 1991 to 2021. The meta-regression analysis was based on 230 estimates of fresh stallion sperm PMOT, analysed through objective CASA systems. Given differences observed in CASA settings, VAP and STR threshold values were included in statistical analyses. Year of semen collection utilised within the analysis ranged from 1984 to 2019. Stallions ranged from 2 to 30 years of age. Breeds analysed included warmbloods (*n* = 64), mixed breeds (*n* = 72), miniature and pony types (*n* = 20), hot-bloods (*n* = 5), and cold-bloods (*n* = 2). There was a higher percentage of stallions ‘unselected for fertility’ (*n* = 151) than selected (*n* = 79). A higher proportion of articles were associated with western populations (*n* = 191) compared to non-western populations (*n* = 32). For the northern hemisphere (total studies *n* = 197), articles from stallions in the breeding (*n* = 62), non-breeding (*n* = 18), and year-round seasons (*n* = 13) were included. For the southern hemisphere (total studies *n* = 26), articles from stallions in the breeding (*n* = 9), non-breeding (*n* = 2), and year-round seasons (*n* = 1) were included.

### Simple linear model

**Figure 2:**
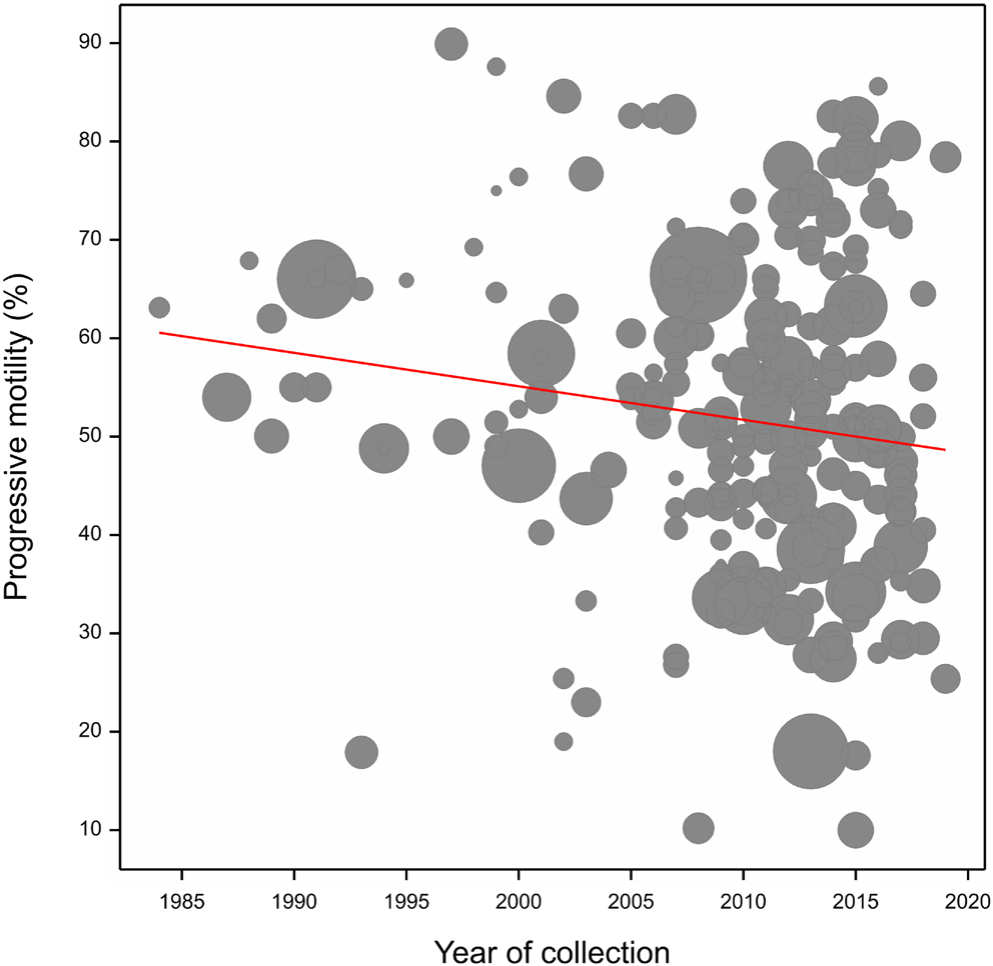
Trends in progressive motility as predicted from the simple linear regression model weighted by sample size. Each data point represents an article included in the assessment. Data point size corresponds to sample size; red line denotes the regression slope.

The simple linear regression model indicated that PMOT declined over the study period (*b* −0.340; *P* = 0.017; Fig. 2). PMOT declined by a value of 11.90% (1984: 60.50 ± 4.04%, 2019: 48.60 ± 1.81%), a yearly and total decrease of 0.56 and 19.67%, respectively.

### Meta-regression models

**Figure 3:**
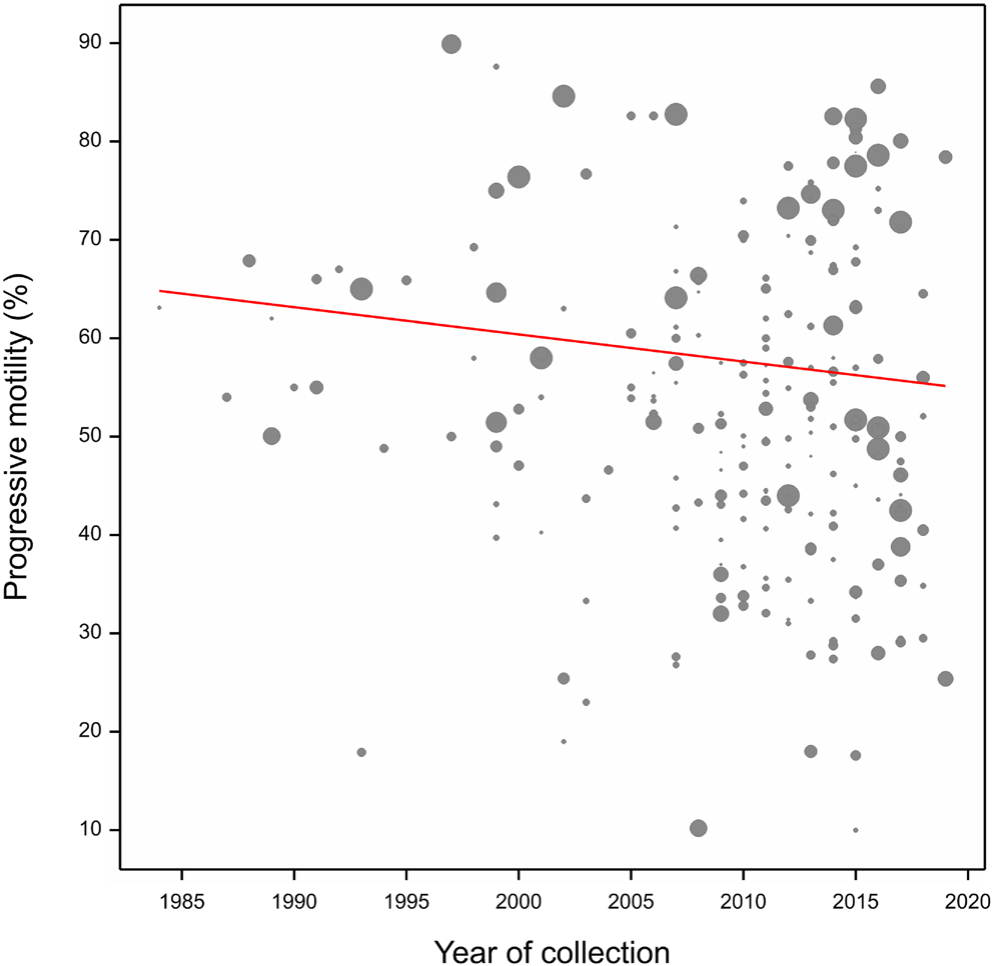
Trends in progressive motility between the years 1984 and 2020, as predicted from the full-refined meta-regression model. Each data point represents an article included in the assessment. Data point size corresponds to sample size; red line denotes the regression slope.

Within the full-refined meta-regression model, accounting for other covariates, PMOT declined significantly (*b* −0.610; *P* < 0.001; Fig. 3). Year of collection (*P* < 0.05), location (*P* < 0.001), hemisphere (*P* < 0.001), stallion breed (*P* < 0.001), and CASA model (*P* < 0.001) were significant within the final refined meta-regression model. Whilst accounting for other covariates, removing the year covariate had a significant effect on the model (*P* < 0.001). PMOT declined by a value of 21.34% (1984: 63.69 ± 5.07%; 2019: 42.35 ± 3.69%), a yearly and overall decline of 0.96% and 33.51%, respectively. The refined model accounted for 47.40% of variance.

### Sensitivity analyses

Multiple analyses were completed to assess the sensitivity of the results and determine the influence of covariates, missing data, and study period. Cubic and quadratic functions added to the year of the collection were insignificant for the meta-regression model (quadratic: *P* > 0.05; cubic: *P* > 0.05; *P = *0.284), so a linear relationship was accepted. The decline and slope of regression did not change appreciably when restricting the analysis to articles with sample sizes of more than 10 stallions (year of collection *P* < 0.05; *b* −0.609; *P* < 0.001; *n* = 73). Restricting the meta-regression analysis to articles with full datasets (*n* = 22) led to a steeper decline in PMOT (year of collection *P* < 0.001; regression: *b* −1.656; *P* < 0.001). In restricting the analysis to collection years 2000–2019 (*n* = 203), PMOT declined significantly (year of collection *P* > 0.05; regression: *b* −0.486; *P* < 0.001). Whilst variability in PMOT between the year of the collection was insignificant (*P* > 0.05), the overall decline was significant (*P* < 0.001). In stallions unselected for fertility potential, PMOT declined significantly between 1984 and 2019 (year of collection *P* < 0.001; *n* = 151; *b* −0.652; *P* < 0.001). PMOT declined by a value of 22.83%, an overall and yearly percentage decline of 36.42% and 1.04%, respectively.

A significant decline was determined in western (year of collection *P* > 0.05; *n* = 191; *b* −0.466; *P* < 0.001) and non-western (year of collection *P* > 0.05; *n* = 32; *b* −0.710; *P* < 0.001) populations. Whilst the year of the collection was insignificant (*P* > 0.05) in both models, overall PMOT declines were significant (*P* < 0.001). In western populations, the predicted overall decline and yearly decline from 1984 to 2019 were 26.29 and 0.75%, respectively (Table 2). Predicted means for non-western populations are presented from 1995 to 2017, with an overall and yearly decline of 10.65 and 0.46%, respectively. For a more accurate comparison between western and non-western populations within an equivalent time scale, western means and trends were predicted in relation to the respective dates reported in the non-western analysis. From 1995 to 2017, the predicted overall decline and yearly decline for western populations were 18.01 and 0.82%, respectively, a steeper decline compared to non-western populations.

**Table 2:** Predicted meta-regression outputs.

Analysis group	n*	First year	First year PMOT**	Final year	Final year PMOT**	Overall change	Yearly change	Slope (95% CI)
Full-refined model	229	1984	63.69%	2019	42.35%	−33.51%	−0.96%	−0.610
>10 Stallions	73	1989	71.31%	2018	53.66%	−24.75%	−0.85%	−0.609
Complete dataset	22	2000	92.48%	2018	34.50%	−45.20%	−2.38%	−1.656
Date restricted	203	2000	51.21%	2019	41.99%	−18.00%	−0.95%	−0.486
F-unselected	151	1984	62.68%	2019	39.85%	−36.42%	−1.04%	−0.652
Western	190	1984	62.04%	2019	45.73%	−26.29%	−0.75%	−0.466
Non-western	32	1995	57.01%	2017	50.94%	−10.65%	−0.46%	−0.710

*Number of articles included in each analysis; **predicted from the fitted model.

Full-refined (meta-regression) model; >10 stallions: articles with >10 stallions; complete dataset: studies with complete datasets; date restricted: studies from 2000 to 2019; F-unselected: fertility unselected.

One article contributed two individual values to the dataset, whilst the remainder only contributed one (229 original articles; 230 data points). Potential skewing, resulting from the inclusion of two values from a single population of stallions, was analysed; however, the slope of the regression was not altered appreciably when investigated individually (*b* −0.653, *P* < 0.001; *b* −0.662, *P* < 0.001; and *b* −0.643, *P* < 0.001, respectively).

## Discussion

This comprehensive evidence synthesis is the first study to report trends in PMOT in global domestic equine populations. Accounting for multiple predetermined covariates, PMOT declined significantly between 1984 and 2019, with an overall and yearly decline of 33.51 and 0.96%, respectively. Adverse PMOT trends within this equine population are of serious concern for the global breeding sector. Given that stallion reproductive function is fundamental in the ability to produce successful progeny, results are concerning for the sustainability and economy of the wider equine industry.

Consistency in trends through multiple sensitivity analyses provides robust evidence to support declining PMOT in equine populations. Adverse trends in PMOT suggest that a higher proportion of stallions are at risk of reduced testicular function, a serious economic (Shakeel *et al.* 2022), health, and welfare concern for the breeding industry. PMOT is significantly correlated with the fertilisation rate *in vitro* (Simon & Lewis 2011). In cooled equine semen, the threshold PMOT value for average and high embryo recovery rates was reported at 45% (Love *et al.* 2015). In the current study, predicted PMOT means in 2019 were below the 45% threshold for high embryo recovery rates. In fresh semen, fertility levels are reported to drop when PMOT values fall below 40% (Colenbrander *et al.* 2003). Whilst PMOT did not drop below this threshold, the value of 39.85% was reported in 2019 for stallions unselected for fertility, a concerning finding given that this subgroup is applicable to wider populations.

Suboptimal PMOT values are likely to put greater strain on sires with desirable genetic traits, which may result in compromised health and welfare associated with industry breeding practices (Campbell & Sandøe 2015). Whilst PMOT declined significantly between 2000 and 2019 in the date-restricted analysis, trends were not as prominent when compared to the full model, which could suggest that the decline in sperm quality is levelling off. A beneficial aspect of utilising publicly available datasets is the ability of research groups to build upon previous reviews. The update of this systematic review will enable continual tracking of trends in PMOT to maintain applicability to current equine breeding statuses. The progression of evidence synthesis is clearly presented in human-based studies (Carlsen *et al.* 1992, Swan *et al.* 2000, Levine *et al.* 2017, 2022).

The debate surrounding declining semen quality has remained controversial since the first human-based evidence synthesis published in 1992 (Carlsen *et al.* 1992). Whilst human evidence syntheses are based on sperm counts as opposed to PMOT, the decline in the current study adds weight to the debate surrounding adverse sperm trends. The current evidence acts as a novel study analysing trends in PMOT through this methodological approach across species, adding to the importance of the findings. The initiation of the equine model as a novel sentinel for the assessment of human-based reproductive trends is a significant outcome of this study. The dog is an established sentinel species for human reproductive trends (Sumner *et al.* 2020), with research presented here reporting a comparable sperm motility decline to that of the canine (Lea *et al.* 2016, Harris *et al.* 2022). Such results support the use of both the established dog sentinel and novel equine model in discussions surrounding declining semen quality trends in humans.

Equine industry practices associated with breeding stallions provide a sampling population that is more randomised compared to human-based research in semen quality trends, which is critiqued for basing results on specific cohorts (Fisch 2008). For the current study, meta-data were collected from stallions presenting for breeding soundness examinations and collections for artificial reproductive technologies. Both breeding practices have become routine within the equine sector (Kowalczyk *et al.* 2019) and the samples selected are likely to be more reflective of greater equine populations. This hypothesis was supported in the statistical analyses of the present study. Individuals unselected for fertility potential are more representative of wider populations (Levine *et al.* 2017), with an equivalent decline reported in this group compared to the full dataset. Selection bias, a pitfall of evidence syntheses (Merzenich *et al.* 2010), eliminated studies that removed individuals based on semen quality, to further ensure data were representative of wider populations. Together, this would suggest that the equine species holds high importance as a comparative model for human trends in reproductive parameters.

Variability of trends in the current study was also equivalent to those reported in humans from a geographical perspective (Levine *et al.* 2017). Results presented here indicate that the decline was more significant in western compared to non-western equine populations. Whilst an equivalent decline was calculated for western vs non-western populations, the reduced sample size for non-western compared to western could act as a potential limitation of the subgroup analysis, nonetheless, a sharper decrease is observed in this instance for westernised regions. Given that an English language restriction was placed on the search strategy, it is also possible that language restrictions based on the geographic origin of the articles included could have impacted the geospatial differences reported both here and in previous literature (Levine *et al.* 2017). The equivalent categorisation method between the current study and previous human-based research (Levine *et al.* 2017) promotes cross-species comparisons, with results indicating that the decline in both equine and human semen quality is more significant in western compared to non-western populations. However, whilst this method of geographic categorisation is accepted in previous evidence syntheses, further variations may exist on national or regional levels.

Differences exist in human motile sperm count between populations in regions of Africa, Asia, the USA, Australia, and Europe (Feferkorn *et al.* 2022). In areas of the USA, a significant geographic-sensitive decline was reported in human sperm motility (Chang *et al.* 2018). Authors attributed the geographic sensitivity to anthropogenic environmental chemicals (ECs), although additional factors that may impact geospatial differences in human populations include overall health, genetics (Sharpe 2010), socioeconomics, nutrition, and lifestyle (Kumar & Singh 2015). Many human-specific factors do not impact reproductive trends in animal species. An equivalent decline in sperm motility in both the established dog sentinel (Lea *et al.* 2016) and the newly proposed equine model is consistent with the hypothesis of an underlying environmental aetiology, highlighting the need for a One Health approach.

Perturbed sperm quality in alternative species is associated with exposure to anthropogenic ECs (Skakkebaek *et al.* 2016), although a link to equine reproductive health has not been evaluated. Given the association of ECs, industrialisation, and perturbations in reproductive parameters, it is plausible that discrepancies in trends reported within the current evidence are linked to geographical variations in contaminant exposure. In China, an area driven by rapid industrialisation, a 3.3% reduction in fertility rate was associated with every 10 µg/m^3^ increment of ambient fine particle pollution (Xue & Zhu 2018). Geographic differences were reported in sperm motility in human populations from the USA, with associations between poor sperm quality and elevated urinary concentrations of alachlor and diazinon, two commercial pesticides (Swan 2006). In the dog sentinel model, whilst geospatial differences in declining reproductive parameters have not yet been analysed (Lea *et al.* 2016), regional differences in testicular EC contamination were associated with geospatial variability in testicular pathologies (Sumner *et al.* 2021). Given the association of poor testicular function and EC exposure in alternative species, further analysis of contaminant interactions with equine reproductive function is warranted. Whilst initiating investigations into the aetiological involvement of ECs and declines presented here requires elucidation, additional species-specific factors must be considered when interpreting trends.

Whilst the analyses of this study include a significant volume of meta-data, the stringent quality appraisal of this systematic review resulted in the removal of half of the available articles. It is important to acknowledge that the utilisation of differing quality appraisal schemes may impact the final selection of articles and could impact the results presented. Whilst this is an inherent limitation of evidence syntheses, ensuring consistent and stringent appraisal methods are utilised is essential in producing accurate results from the data generated. Furthermore, the impact of inbreeding depression on adverse PMOT trends reported within the current study cannot be overlooked. High inbreeding coefficients are associated with poor semen quality including motility in multiple equine breeds with restricted gene pools (Van Eldik *et al.* 2006, Ducro *et al.* 2014, Pirosanto *et al.* 2020, 2021, Dini *et al.* 2020). In Thoroughbreds, a breed at high risk of inbreeding depression (McGivney *et al.* 2020), whole genome sequencing (*n* = 150) suggested no association with fertility (Castaneda *et al.* 2021), indicating that there is not a definitive link. Given that adverse trends in sperm motility characteristics are reported across species (Lea *et al.* 2016, Tiegs *et al.* 2019), it is likely that a common aetiology exists other than species-specific factors alone. Whilst breed is not a direct measure of inbreeding, it was accounted for within the statistical model. Considering the method of breed categorisation, stallions were grouped using recognised methods (Ebel *et al.* 2020). However, differences in sperm trends may be on a more specific level, indicating a potential limitation of the broad categorisation methods required in this data set.

Accounting for abstinence or age remains a limitation of this style of research (Fisch 2008). Whilst calculating the year of semen collection from the publication date is an accepted systematic method (Levine *et al.* 2017), reduced accuracy remains a concern (Boulicault *et al.* 2021). Here, only 22 articles provided full datasets, limiting the conclusions that can be taken from the restricted analysis. Utilising retrospective datasets providing accurate data on timings for individual collections would mitigate this limitation, highlighting the need for further research in single equine populations. Furthermore, CASA systems can impact the readings of sperm motion characteristics (Whitesell *et al.* 2020), so it is recommended that settings are accounted for when comparing data from multiple laboratories (Hu *et al.* 2013). Within the current meta-regression, VAP and STR were not significant within the model and, despite graphically presenting slight adverse trends, are unlikely to be responsible for declines reported in PMOT across the study period. CASA model and settings were accounted for where possible, however advances in semen analysis methods of this technology, and in general semen laboratory techniques, could have impacted the results, which is an inherent limitation of studies analysing semen quality trends. Standardisation of CASA parameters specific to the horse would benefit the consistency and interpretation of results in motility and kinematic parameter measurements, enabling further comparative research in stallion sperm quality. Transparent and consistent reporting of CASA models and settings utilised for equine sperm analysis is required to further mitigate the limitations associated with collecting and analysing data through systematic approaches.

## Conclusions

These data show a significant PMOT decline in global domesticated equine populations between 1984 and 2019. Adverse trends in PMOT imply that an increasing proportion of stallions are at risk of perturbed reproductive health and function, with serious implications for the economic status of breeding stallions, and the health and welfare of breeding stock. The novel equine model has contributed significant data to the debate surrounding declining fertility in human populations, proving an invaluable comparative model for further research in the reproductive biology sector. Further interdisciplinary research enabling cross-species comparisons and a One Health approach should be employed to further elucidate the aetiologies underlying adverse trends across species.

## Declaration of interest

The authors declare that there is no conflict of interest.

## Funding

This study did not receive any specific grant from any funding agency in the public, commercial or not-for-profit sector.

## Author contribution statement

RNB, ITH and AZP conceived the study. RNB, ITH, AZP, MF, and KN designed the review. CM and JP acted as independent reviewers for article screening. ITH analysed, interpreted the data, and produced the figures. ITH and RNB wrote the manuscript.
